# Comparing Postoperative Leg Length Discrepancy and Femoral Offset Using Two Different Surgical Approaches for Hemiarthroplasty of the Hip

**DOI:** 10.51894/001c.25096

**Published:** 2021-08-30

**Authors:** James T. Layson, Matthew S. Coon, Rajan Sharma, Benjamin Diedring, Alan Afsari, Benjamin Best

**Affiliations:** 1 Orthopaedic Surgery Ascension Macomb Oakland Hospital; 2 Orthopaedic Surgery Ascension St. John Hospital

**Keywords:** orthopaedic surgery, leg length discrepancy, direct anterior approach, anterolateral approach, hemiarthroplasty

## Abstract

**INTRODUCTION:**

The direct anterior approach (DAA) and anterolateral approach (ALA) may be used for hip hemiarthroplasty (HHA) as a treatment for femoral neck fractures. The DAA often utilizes intraoperative fluoroscopy to determine leg length and offset, while the ALA traditionally utilizes an intraoperative clinical exam to determine offset and leg length. This study will evaluate two techniques: the “grid fluoroscopy [GF] technique” and the “intraoperative exam [IE] technique,” each performed by one of two separate surgeons, and compare each technique’s accuracy to restore leg length and femoral offset in a patient population that underwent HHA.

**METHODS:**

Two investigators retrospectively reviewed charts of 208 randomly selected patients who had an HHA from either a DAA or ALA performed by two different surgeons for the treatment of femoral neck fractures. Postoperative AP pelvis radiographs were measured to determine offset and leg length compared with the non-operative extremity. Non-normal continuous variables were provided by median and interquartile range. Data were analyzed with the Mann-Whitney U test and Student’s t-test.

**RESULTS:**

After inclusion and exclusion criteria, data were reviewed on 173 hemiarthroplasties. The mean age was 80.3 years (± 11.2 years). Of the surgical patients, 65.9% were female, and 70.9% identified their ethnicity as white. The DAA was used in 93 patients and ALA in 80 patients. Analysis comparing the two techniques demonstrated no statistically significant differences in median leg length between GF technique (1.02 IQR -0.1, 2.0 mm) and IE technique (1.25 IQR -2.4, 1.3 mm,) (p=0.67). There was also no statistically significant difference in offset between GF technique (1.3 IQR 0.2, 2.1 mm) and IE technique (0.6 IQR -2.7 mm, 3.2 mm) (p=0.13). However, a difference was found in mean length of surgery that was statistically significant. We found that the mean length of surgery for the IE technique was 74.8 ± 24.7 minutes versus the GF technique, which was 95.1 ± 23.0 minutes, (p<0.0001).

**DISCUSSION:**

There was no significant difference between leg length and offset with the use of intraoperative fluoroscopy with DAA compared to no intraoperative imaging with ALA. Our study suggests that DAA and ALA are equally effective approaches for re-establishing symmetric leg length and offset in HHA for femoral neck fractures. In this study, the ALA had a shorter surgical time compared to DAA, potentially due to the utilization of intraoperative fluoroscopy for this particular technique during the DAA.

## Introduction

Hemiarthroplasty has become the mainstay of treatment for elderly patients with a displaced femoral neck fracture with a low conversion rate to total hip arthroplasty in the elderly population.^1^ Restoration of hip biomechanics is an essential component of successful arthroplasty.^2–4^ During arthroplasty, the reestablishment of femoral neck length and femoral offset, which is the distance between the femoral head center of rotation and a line drawn down the axis of the femoral shaft, is a critical portion of the case, and directly influences hip biomechanics.^2–4^ Restoration of these measurements can affect surrounding anatomic tension-altering biomechanics, and more directly, patient function.^2,3,5–8^

**Figure 1.**
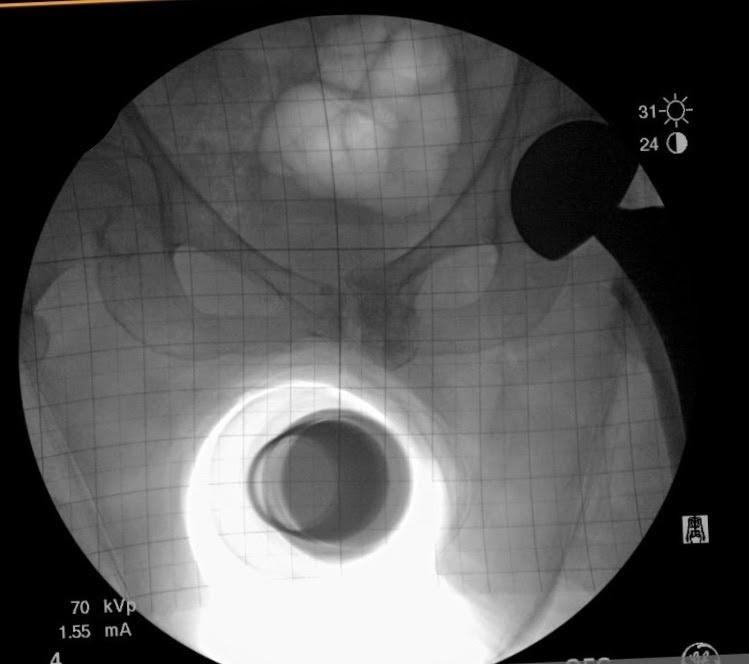
Intraoperative fluoroscopic imaging used in the grid technique

Multiple surgical approaches have been described for hip hemiarthroplasty (HHA) in the setting of a displaced femoral neck fracture.^9^ The direct anterior approach utilizes the intermuscular and internervous plane between the sartorius (i.e., hip flexor in the anterior thigh innervated by the femoral nerve) and tensor fascia latae (i.e., hip abductor innervated by the superior gluteal nerve) superficially, and the rectus femoris (i.e., hip flexor and knee extensor in the anterior thigh innervated by the femoral nerve) and gluteus medius (i.e., hip abductor innervated by the superior gluteal nerve) for the deep layer.^10^ This approach is typically performed in the supine position, which allows the use of intraoperative fluoroscopy, and in our study, a grid to measure and assess implant positioning, monitoring for leg length discrepancies (LLD) and femoral offset (FO) (Figure 1a). In contrast, the anterolateral approach utilizes the intermuscular plane between the tensor fascia latae and gluteus medius. The patient is positioned on a non-radiolucent peg board in the lateral position, which does not allow for utilization of a fluoroscopic grid.

Multiple techniques have been proposed and utilized for accurate restoration of hip biomechanics and measurements in total hip arthroplasty (THA) and HHA.^2,11–13^ The literature is scarce comparing these techniques for accurate restoration in hip hemiarthroplasty for displaced femoral neck fractures. This study will evaluate two techniques: the “grid fluoroscopy [GF] technique” and the “intraoperative exam [IE] technique,” each performed by one of two separate surgeons, and compare each technique’s accuracy to restore leg length and femoral offset in a patient population that underwent HHA.

## Methods

A retrospective collection of 200 hemiarthroplasties for acute femoral neck fractures were performed, with the goal to measure differences between postoperative leg lengths and femoral offset. One hundred patients were collected for each surgeon to compare the techniques described below. These surgeries were performed at a single institution by two trauma-trained Orthopaedic surgeons. Patient data during the time period between 2008-2019 was reviewed for the study. This time period was chosen to encompass the entire collection period of hip fractures between the two surgeons at the involved institution.

Among criteria for inclusion in the study, patients had to be over the age of 65 sustaining a hip fracture requiring hemiarthroplasty (Garden 3 or 4 – complete femoral neck fracture with partial or complete displacement respectively). Exclusion criteria involved patients younger than 65, patients with an ipsilateral intraoperative fracture, known preexisting ipsilateral chronic hip deformity, patients with a previous contralateral intramedullary antegrade femoral nail (i.e., an orthopaedic implant consisting of a rod and screw commonly used to treat hip fractures that do not require hip hemiarthroplasty), and patients with previous contralateral hip arthroplasty, due to potential changes in native hip length and offset for comparison. We did not exclude contralateral hip arthritic changes or deformities due to the subjective nature of these deformities. The data collected included patient age at the date of surgery, BMI, gender, race, mechanism of injury, fracture classification, surgeon, approach and technique, LLD, femoral offset, and length of the procedure (minutes). The data were collected and stored electronically on a hospital network, encrypted, and password protected. Patient identifiers were removed for confidentiality and given unique study identifiers.

**Figure 2.**
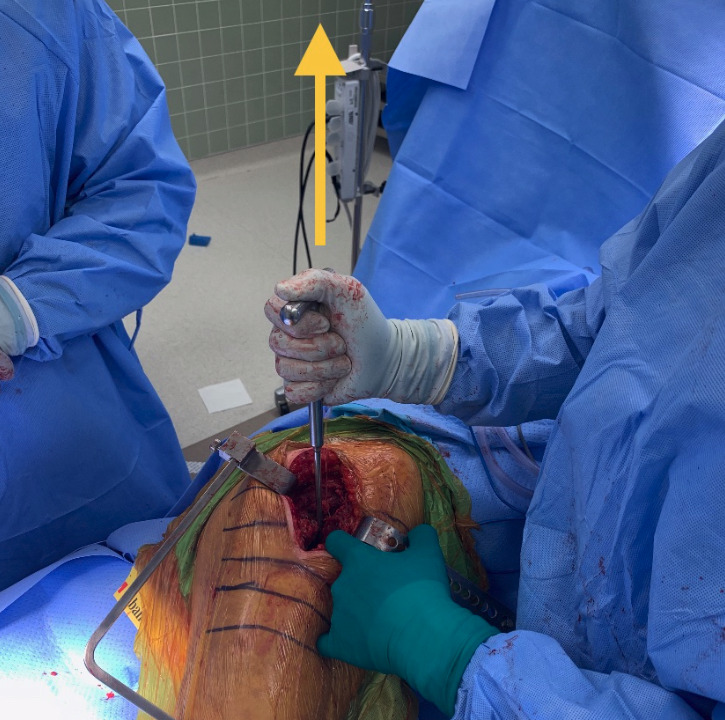
Abductor shuck test, with bone hook elevating the hip to assess overall fit and tissue tension

At our institution, both surgeons each have their own technique, which has remained unchanged over the last eight years. One surgeon, using the GF technique, performs a supine, direct anterior approach with intraoperative fluoroscopic imaging utilizing a grid to view the internal components and to estimate the leg length and offset compared to the contralateral extremity (Figure 1a). The other surgeon, using the IE technique, uses an anterolateral approach with no intraoperative imaging. In order to reestablish neck length, the surgeon judges the length by visualizing and attempting to replace the amount of bone that was removed above the lesser trochanter (i.e., a bony anatomic landmark located near the hip). To restore offset, a manual intraoperative abductor shuck test is performed. For this test, the size of the femoral head is measured and used as a guide for the trial size for testing. After the hip hemiarthroplasty trial is reduced, soft tissue tension is manually assessed. Next, the surgeon applies a distracting force with a bone hook around the neck of the implant to check for overall fit and suction within the acetabulum (i.e., the socket of the hipbone where the head of the femur fits) (Figure 2). The soft tissue tensioning of the surrounding hip musculature is judged by whether the abductor musculature can be repaired without excessive tensioning. Leg length is again reassessed intra-operatively by comparing the medial malleoli (i.e., the inner side of the ankle) and patella (i.e., the small bone located in the front of the knee joint) of each leg.

**Figure 3.**
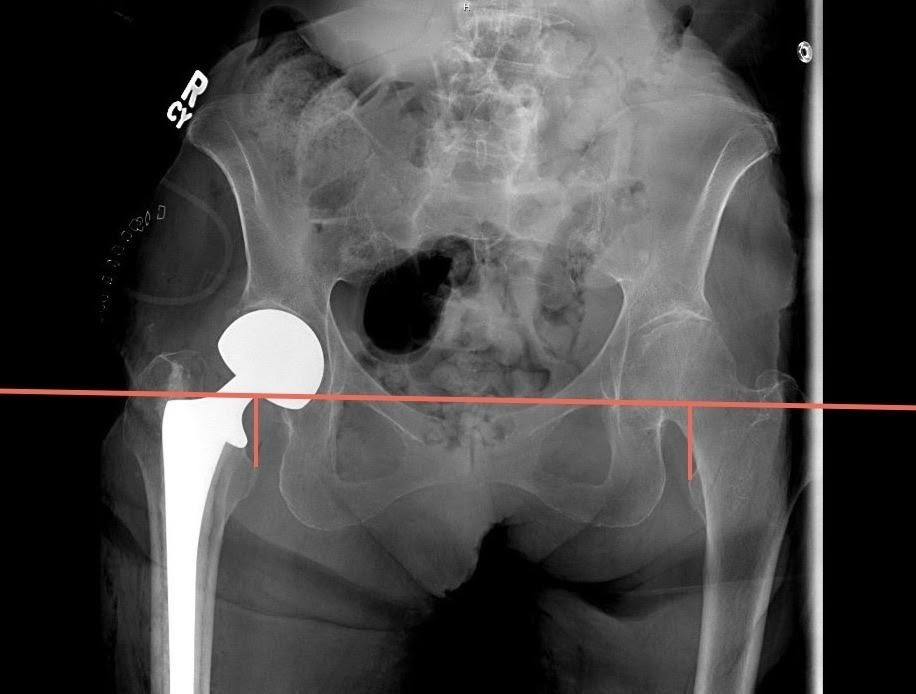
Technique used to measure comparative postoperative leg length

Measurement of LLD was performed using immediate postoperative imaging of an AP pelvis radiograph utilizing the technique described by Ranawat et al.^14^ This measurement was performed using the difference between a horizontal line drawn from the inferior aspect of the teardrop of the pelvis to a horizontal line drawn from the most prominent aspect of the lesser trochanter. Positive values were given if the length on the operative side is greater than the contralateral side, and negative values were given if the operative side was shorter (Figure 3).

**Figure 4.**
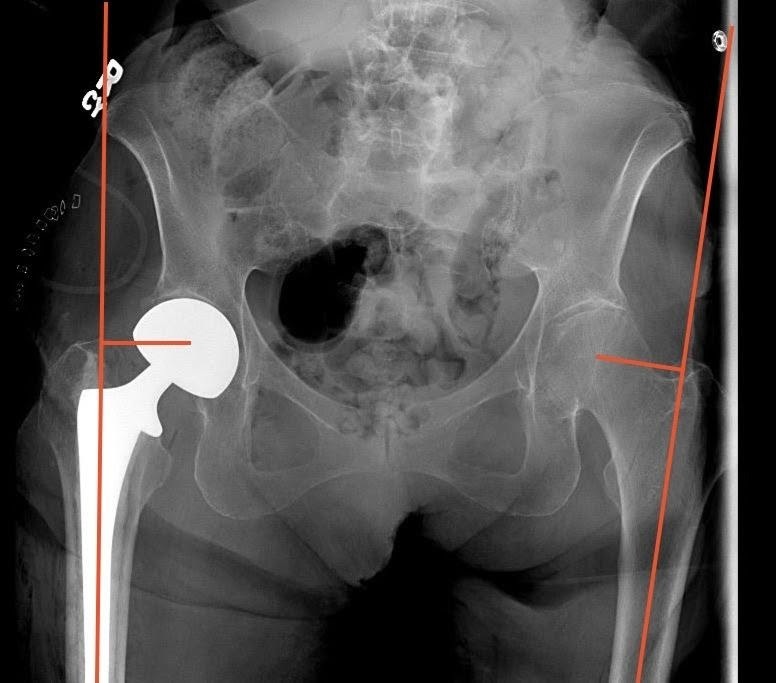
Technique used to measure comparative postoperative hip offset

Measurement of FO was also performed using immediate postoperative imaging of an AP pelvis radiograph utilizing the technique described by Asayama et al.^15^ This technique measures the distance from a vertical line drawn from the center of the femoral head to a line bisecting the femoral shaft. Positive values were given if the operative side had a greater offset than the contralateral side, and negative values were given if the operative side had less offset (Figure 4).

Measurements were taken by a group of orthopedic residents using the same technique. Inter-examiner variability was assessed by a random sample of 10 patients from each type of surgery being measured by each resident, blind to the other resident’s measurements. Intra-examiner variability was established by each investigator re-measuring a random sample of 10 patients after enough time has elapsed, so measurements are not remembered and blind to the previous measurements.

Descriptive statistics were calculated for the study group. Continuous, normally distributed variables were described as the mean with standard-deviation; non-normally distributed continuous variables were described as the median with interquartile range. Univariable analysis was done using the chi-squared test, Student’s t-test and the Mann-Whitney U test. All data were analyzed using SPSS v.26.0 (IBM, Armonk, NY) and a p-value less than 0.05 was considered to indicate statistical significance (SS, please see acknowledgements).

## Results

After inclusion and exclusion criteria, data were reviewed on 173 hemiarthroplasties. The mean age was 80.3 years (SD = 11.2 years). Of the surgical patients, 65.9% were female, and 70.9% identified their ethnicity as white. The DAA was used in 93 patients and ALA in 80 patients. A table summarizing key data is noted in table form (Table 1).

**Table 1.** A Comparison of Baseline Characteristics Between Groups

*Variable*	*Anterior (n = 93)*	*Anterolateral (n = 80)*	*P-value*
			
*Age (years)^a^*	81.6 (10.7)	79.1 (11.7)	0.14
			
*Female Gender^b^*	64.5%	66.3%	0.81
			
*BMI^a^*	24.14 (5.5)	25.0 (4.7)	0.29
			
*Length of Surgery (minutes)^a^*	95.1 ± 23	74.8 ± 24.7	P < 0.0001

^a^Student t-test, mean (standard deviation).

^b^Chi-squared analysis, % (n).

BMI, body mass index.

Analysis comparing the two techniques demonstrated no statistically significant differences in median leg length between GF technique (1.02 IQR -0.1, 2.0 mm) and IE technique (1.25 IQR -2.4, 1.3 mm,) (p = 0.67). There was also no statistically significant difference in offset between GF technique (1.3 IQR 0.2, 2.1 mm) and IE technique (0.6 IQR -2.7 mm, 3.2 mm) (p = 0.13). However, a difference was found in the mean length of surgery that was statistically significant. We found that the mean length of surgery for the IE technique was 74.8 (SD = 24.7 minutes) versus the GF technique, which was 95.1 (SD = 23.0 minutes), (p < 0.0001). (Table 1) This is potentially due to the utilization of intraoperative fluoroscopy for this particular technique during the DAA. A table summarizing additional key measurement results are below in table form (Table 2).

**Table 2.** Radiographic Outcomes

*Variable*	*Anterior (n = 93)*	*Anterolateral (n = 80)*	*P-value*
			
*Leg length (mm)^a^*	1.1(-0.1, 2.0)	1.25 (-2.4, 4.2)	0.68
			
*Offset (mm)^a^*	1.3 (0.2, 2.1)	0.6 (-2.7, 3.2)	0.12

^a^Mann-Whitney U test, median (interquartile range).

## Discussion

As previously discussed, restoration of the leg length and femoral offset compared to the contralateral side is an important component for surgical outcome.^2–4,16,17^ In hemiarthroplasty, Ji et al. demonstrated that in patients with femoral offset differing more than 20%, the preoperative values had significant correlation with worse Modified Barthel Index (MBI) scores (measurement of dependence), but not Harris Hip Scores (HHS) (patient reported outcomes after hip arthroplasty).^5^ Buecking et al also found a positive correlation between femoral offset and HHS.^4^ Leg length discrepancy <10 mm has been shown to have clinically acceptable patient satisfaction.^18,19^ Edeen et al. showed that leg length inequality has shown high rates of dissatisfaction and was correlated with abnormal gait and use of ambulatory assistive devices.^20^

It has been shown previously that fluoroscopy use during the anterior approach can improve component positioning, notably acetabular cup position.^11,21,22^ Hasegawa et al published a study using intraoperative fluoroscopy and a grid system without any preoperative templating and had no differences in offset or leg length greater than 10 mm.^12^ Other techniques have been utilized, including using intraoperative fluoroscopic references to the contralateral side with a radiopaque line^23^ as well as comparing an intraoperative radiograph to preoperative contralateral radiograph overlay.^24,25^

Certain surgical techniques are not without their downsides. Fluoroscopy is commonly used along with some kind of radiopaque marker to compare leg length to the contralateral side. With fluoroscopy comes the potential for radiation exposure to the surgeon, as well as the patient.^22^ McArthur et al. looked at radiation levels in 51 primary total hip arthroplasty and found that the average fluoroscopic time was 0.59 minutes.^21^ Other studies showed that patient radiation levels were between 1.78-3 mGy. Though this number varies and is relatively low, (3.1 mGy is the average annual background radiation in United States)^26,27^ it is important to understand especially if performing large volumes of these cases.

Our study compared two different techniques and their efficacy against one another in restoring leg length and offset. Analysis demonstrated that there were no statistically significant differences between LLD or FO between the two techniques. This demonstrates that both techniques are equally effective, and perhaps what matters is a cognizant attempt to restore leg length and FO. The study did however show that the IE technique had a significantly shorter average surgical time of approximately 17 minutes. This significance may be due to the use of intraoperative fluoroscopy during the GF technique utilized in this specific DAA. Because of these differences and the lack of other variable controls such as type of implant, press-fit versus cemented, and resident participation, this may not be generalizable, but speaks to the difference between these two specific techniques.

Our study is not without limitations. This study involved a single center with two trauma surgeons. These surgeons used not only a different technique to restore leg length and FO, but they also used different surgical approaches. We acknowledge that this creates a possible confounding variable trying to compare the two techniques of intraoperative assessment of leg length and FO, especially in regard to surgical time. Our study also looked at only LLD and FO in postoperative X-rays, in which the rotation of the hips was not controlled by the technician, leading to potential measurement discrepancies. Another potential weakness is that the type of anesthesia (general versus spinal) was not controlled, as this was left up to the anesthesia team’s discretion, however this should be similar between the two groups. Also, the intraoperative paralysis at time of testing was not controlled, which could have potentially led to differences in implant size selection and soft tissue tensioning. This study did not look at the outcomes associated with each technique, such as the HHS or the MBI. This study was to specifically assess the objective parameters comparing the two techniques. Future studies comparing functional outcomes may be needed to further evaluate these additional concerns.

Power analysis was also performed prior to collection, using the study by Bingham et al as a guide.^28^ To find a difference between a mean of 1.0 ± 0.5 and 0.8 ± 0.5, with 80% power and an alpha error rate of 0.05, we determined the study would require 100 subjects per group. Though that was the aim of this study, there were limitations to provide the total number required, including but not limited to, the lack of adequate postoperative imaging, existing contralateral arthroplasty, existing contralateral intramedullary nails, and the total number of patients at the institution fitting inclusion criteria. Some of these limitations can be avoided in future studies by ensuring that all patients receive adequate and rotationally controlled postoperative imaging. A multi-institution study may be needed to adequately obtain the necessary power for future studies.

The authors believe that further studies comparing these techniques could prove to be beneficial. A larger sample of patients would be preferred to see the magnitude of the effects. Also, a control of the surgical approach may also provide insight into the potential operative time savings of one technique. Finally, further studies may also wish to collect and compare HHS or MBI to compare clinical outcomes of the two techniques.

## Conclusion

This study compared two different intraoperative techniques to restore leg length and FO. There was no statistical difference between LLD or FO between the two groups, suggesting that both techniques were equally efficacious. This study may provide insight that, as long as there is a cognizant attempt to restore leg length and FO, intraoperative fluoroscopy along with use of a grid, is not necessarily required to restore leg length and offset in hip hemiarthroplasty.
